# A Medical Poem

**Published:** 1853-10

**Authors:** Oliver Wendell Holmes


### RESPONSE

BY OLIVER WENDELL HOLMES, M.D.,

*To the following Toast, proposed at the Entertainment given to the American Medical Association, by the Physicians of the City of New York, at Metropolitan Hall, on the 5th of May,* 1853*:*

TOAST.—"The Union of Science and Literature—A happy marriage, the fruits of which are nowhere seen to better advantage than in our American *Holmes*."

I hold a letter in my hand—
A flattering letter—more's the pity—
By some contriving junto planned,
And signed, *per order of Committee;*
It touches every tenderest spot—
My patriotic predilections—
My well known—something—don't what—
My poor old songs—my kind affections:—

They make a feast on Thursday next,
And hope to make the feasters merry—
They own they're something more perplext
For poets than for port and sherry—
They want the men of—(word torn out);
Our friends will come with anxious faces,
(To see our blankets off, no doubt,
And trot us out and show our paces).

They hint that papers by the score
Are rather musty kind of rations:
They don't exactly mean a bore,
But only trying to the patience;
That such as—you know who I mean—
Distinguished for their—what d'y call 'em?—
Should bring the dews of Hippocrene
To sprinkle on the faces solemn.

—The same old story; that's the chaff
To catch the birds that sing the ditties;

Upon my soul, it makes me laugh
  To read these letters from Committees!
They're all *so* loving and *so* fair—
  All for *your* sake such kind compunction—
'Twould save your carriage half it's wear
  To grease the wheels with such an unction!

Why, who am I, to lift me here
  And beg such learned folk to listen—
Ask a smile, or coax a tear
  Beneath these stoic lids to glisten?
—As well might some arterial thread
  To ask the whole frame to feel it gushing,
While throbbing fierce from heel to head
  The vast aortic tide was rushing.

As well some hair-like nerve might strain
  To set its special streamlet going,
While through the myriad-channeled brain
  The burning flood of thought was flowing—
Or trembling fibre strive to keep
  The springing haunches gathered shorter,
While the scourged racer, leap on leap,
  Was stretching through the last hot quarter!

Ah me! you take the bud that came
  Self-sown in your poor garden's borders,
And hand it to the stately dame
  That florists breed for, all she orders;
*She thanks you—it was kindly meant—*
  *(A pale affair not worth the keeping—)*
*Good morning;*—and your bud is sent
  To join the tea leaves used for sweeping.

Not always so, kind hearts and true—
  For such I know are round me beating—
Is not the bud I offer you—
  Fresh gathered for the hour of meeting—
Pale though its outer leaves may be,
  Rose-red in all the inner petals,

Where the warm life we cannot see—
The life of love that gave it, settles?

We meet from regions far away
Like rills from distant mountains streaming:
The sun is on Francisco's bay,
O'er Chesapeake the lighthouse gleaming:
While summer skirts the still bayou
With every leaf that makes it brighter,
Monadnock sees the sky grow blue
And clasps his crystal bracelet tighter.

Yet Nature bears the self-same heart
Beneath her russet-mantled bosom,
And where, with burning lips apart,
She breathes, and white magnolias blossom:
Ay! many a cheek is kindled here
With morning's fire as richly laden
As ever Sultan of Cachemere
Kissed from a sun-enameled maiden!

I give you *Home!* its crossing lines
United in one golden suture,
And showing every day that shines
The present growing to the future—
A flag that bears *a hundred* stars,
In one bright ring, with love for centre,
Fenced round with white and crimson bars,
No prowling treason dares to enter!

O brothers, home may be a word
To make affection's living treasure—
The wave an angel might have stirred—
A stagnant pool of selfish pleasure;
HOME! It is where the day star springs
And where the evening sun reposes,
Where'er the eagle spreads his wings
From Northern pines to Southern roses!

—*Virginia Medical and Surgical Journal.*

